# Indonesian Dentists’ Perception of the Use of Teledentistry

**DOI:** 10.1016/j.identj.2022.04.001

**Published:** 2022-05-12

**Authors:** Anandina Irmagita Soegyanto, Yuniardini Septorini Wimardhani, Diah Ayu Maharani, Marc Tennant

**Affiliations:** a Department of Oral Medicine, Faculty of Dentistry, Universitas Indonesia, Jakarta, Indonesia; b Department of Preventive and Public Health Dentistry, Faculty of Dentistry, Universitas Indonesia, Jakarta, Indonesia; c School of Human Sciences, University of Western Australia, Perth, Australia

**Keywords:** Telemedicine, Teledentistry, Dentists, Perception, Pandemic

## Abstract

**Introduction:**

Teledentistry is the use of information and communication technology to provide dental services from distant locations. The use of teledentistry is highly beneficial in the COVID-19 pandemic era.

**Objective:**

This study aimed to explore Indonesian dentists’ perceptions of the use of teledentistry in their daily practice and the benefits for patients.

**Methods:**

This was a descriptive cross-sectional study comprising an electronic survey of Indonesian dentists. We used a cross-cultural, adapted questionnaire that consisted of 26 items and 5-point Likert scale questions that evaluated dentist perception in the following 4 domains: usefulness of teledentistry for dental practice, usefulness of teledentistry to improve practices, usefulness of teledentistry for patients, and dentists who had concerns about the use of teledentistry.

**Results:**

A total of 652 dentists from 34 provinces in Indonesia participated in this study. The majority of respondents agreed about the usefulness of teledentistry in dental practice, especially for saving time, compared to referral letters (87%). Most respondents recognised the utility of teledentistry for improving dental practice and its benefits for patients. Nevertheless, most of the dentists had concerns about teledentistry in terms of digital forgery concern (74.2%) and technical incompatibility (71.8%).

**Conclusions:**

Indonesian dentists reported their positive perception of the usefulness of teledentistry for improving dental practice and benefits for patients, although some concerns were still present. Further studies on the application of teledentistry in Indonesia are still needed.

## Introduction

Telemedicine is defined as a long-distance health service provision and health information exchange using communication and information technology. Telemedicine includes diagnosis, disease treatment and prevention, continuing education for health care workers and users, research, and evaluation, with the intention of increasing individual and community health.^1^^,^^2^ It has been applied in various medical fields, including dentistry, which is known as teledentistry.^3^ Teledentistry is the provision of dental treatment in real time or offline, such as diagnosis, treatment planning, consultation, and follow-up through electronic transmission from distant locations.^4^, ^5^, ^6^ It enables us to connect and overcome obstacles related to distance by expanding treatment outcomes for populations with inadequate access to care and for general dentists in need of specialty work.^6^^,^^7^ Many studies on teledentistry worldwide have explored its use to perform dental and oral mucosal screening, as a diagnostic aid, for referral and consultation, and for educational purposes.^8^^,^^9^ These studies also included specialist care of dentistry in daily practice, such as caries diagnostic in paediatric dentistry,^10^^,^^11^ orthodontic treatment,^12^^,^^13^ dental trauma,^14^^,^^15^ periodontology,^16^ and oral medicine.^17^, ^18^, ^19^, ^20^, ^21^, ^22^, ^23^

Some studies have shown that the application of teledentistry in daily practice is not always well accepted.^8^^,^^24^ Several aspects have been identified as challenges of teledentistry usage, including information confidentiality related to patient data, informed consent, the risk of misdiagnosis, ethical problems, and financing problems related to consultation services in teledentistry.^8^ Some studies have reviewed patient satisfaction and dentist perceptions of the use of teledentistry in daily practice.^18^^,^[25], [26], 27

The use of teledentistry in Indonesia before the COVID-19 pandemic era has not been widely explored. Several articles related to the use of teledentistry in Indonesia are still limited to the role of electronic medical records and the use of telecommunication media in orthodontic treatment.^28^^,^^29^ During the COVID-19 pandemic, the Indonesian government has encouraged the use of telemedicine^30^^,^^31^ to also be applied in the dental field. The application of teledentistry enables prior triage to aid patient screening effectiveness, prioritise patients’ needs, support self-quarantine measurements, and eventually protect patients, health care workers, and the community from COVID-19 exposure.^32^ Dental personnel are at high risk for being exposed to COVID-19, since dental procedures can potentially create particle and aerosol inhalation from infected patients^33^; therefore, the use of teledentistry is expected to reduce the risk.^24^ The new regulation to use teledentistry would bring some response amongst Indonesian dentists in terms of perceptions of its use. This study aims to explore Indonesian dentists’ perceptions of the use of teledentistry in their daily practice and explore the determining factors that influence their perceptions.

## Methods

### Questionnaire

This study used a questionnaire from previous studies that had been cross-culturally adapted to Indonesians.^26^^,^^27^ The questionnaire consisted of 4 sections. The first section consisted of professional and demographic information. In the second section, there were twenty-one 5-point Likert-type questions divided into 3 domains: capability of teledentistry to improve practice and usefulness of teledentistry for the dental practice and for patients. The third section consisted of five 5-point Likert-type questions about concerns of data and security related to teledentistry. The last section was about dentists’ preferred dental specialties for teledentistry use. The questions were transformed into online versions and pretested with a group of dentists to gain opinion and assess general suitability of the questionnaire. Minimal corrections were applied based on these pretested responses, and the final form of the questionnaire was an online version that was distributed to the participants. An informed consent page was included in the online form, and dentists who agreed to participate could complete the survey. Ethical approval for the study was obtained from the Ethical Committee of the Faculty of Dentistry, Universitas Indonesia, No. 6/Ethical Approval/FKGUI/VI/2020, with protocol No. 070060520.

### Questionnaire distribution

This was a cross-sectional survey-based study using a nonprobability sampling method. The questionnaire was distributed to all dentists, and dentists who completed the questionnaire were included in the study. The questionnaire distribution was facilitated by the Indonesian Dental Association (IDA). The IDA contacted each of the head area divisions throughout 34 provinces in Indonesia to circulate the questionnaire to their members. The questionnaire was distributed to the members using the WhatsApp group. The call for participation in the study was opened for a month. During that time, the invitation to participate in the study was reposted by the IDA head area division to the group once a week.

### Data analysis

The completed response was transferred to IBM SPSS Statistics for Windows version 22 (IBM Corp.) for statistical analysis. Descriptive statistics, including means, standard deviations, and frequency distributions, were performed. The Kruskal–Wallis test was performed to evaluate differences in the mean score of responses for variables such as age, gender, work experience, and qualification when the distribution of data was not normal. A *P* value of .05 was used for statistical significance.

## Results

### Characteristic of study participants

An electronic survey of Indonesian dentists was carried out between January and February 2021. A total of 652 dentists completed the questionnaire. Almost half of the dentists were in the age group 20 to 34 years (46.3%), with the majority being female (76.2%), being general dentists (74.2%), and working in nonremote areas (87.3%). More than a quarter are dentists who have 0 to 5 years of work experience (27.6%), with almost one-third of them working in public health centres (32.2%) and having clinical work 35 to 49 hours per week (44.2%). In terms of Internet use and communication method preferences, this study showed that almost one-third of dentists had an average of 5 to 7 hours of daily Internet use for general purposes (32.4%), whilst its use related to practice was for 2 to 4 hours (44.2%) (Table 1). Most of the dentists in this survey preferred social media as their communication tool (96.01%), with the favoured social media used being the WhatsApp application (98.8%), Instagram (52.5%), and Facebook (28.2%). In terms of smartphone type used by these dentists, Android-type smartphones were more preferred (75.6%) than iOS-type smartphones (24.1%), and most dentists preferred to use applications in their smartphones (86.0%) rather than searching in browsers (13.2%).

**Table 1:** Description of demographics, professional characteristics, internet usage, and preferred communication tools of Indonesian dentists (N = 652).

Characteristic	Frequency	%
**Age (years)**		
20–34	302	46.3
35–44	230	35.3
45–54	86	13.2
55–64	32	4.9
≥65	2	.3
**Gender**		
Male	155	23.8
Female	497	76.2
**Qualification**		
Specialists	113	17.3
General dentists	484	74.2
Residents	55	8.4
**Work experience (years)**		
0–5	180	27.6
6–10	172	26.4
11–15	137	21.0
>15	163	25.0
**Location of main job**		
Not remote	569	87.3
Remote	70	10.7
Very remote	13	2
**Work setting of main job**		
Public health centre	210	32.2
Private clinic	205	31.4
Hospital	166	25.5
Academic	71	10.9
**Working hours per week**		
1–19	137	21.0
20–34	173	26.5
35–49	288	44.2
50–64	38	5.8
>65	16	2.5
**Daily general-purpose use of internet (hours)**		
<1	15	2.3
2–4	198	30.4
5–7	211	32.4
8–10	107	16.4
>11	121	18.6
<1	15	2.3
**Daily practice-related use of internet (hours)**		
<1	221	33.9
2–4	288	44.2
5–7	85	13.0
8–10	32	4.9
>11	26	4.0
**Preferred communication tools**		
In person	258	39.57
By phone	332	50.92
Text message	62	0.95
Letters	3	0.46
Email	81	12.42
Social media	626	96.01
Video conference	147	22.55

### Usefulness of teledentistry for dental practice

This domain consisted of 7 Likert-type questions that evaluate dentist perception of the usefulness of teledentistry for dental practice. The mean total score of this domain was 23.27 ± 2.364, and the mean Likert score of this domain was 3.32 ± 0.34. Most respondents agreed that teledentistry is useful for dental practice in terms of providing adequate diagnostic information (58%), saving time compared to referral letters (87%), reducing costs for dental practice (63%), and enhancing continuing education (57%). However, almost half of the respondents were not sure whether teledentistry would be too expensive to set up (47%), as shown in Table 2.

**Table 2:** Dentists’ perception of teledentistry usefulness for the benefit of the dental practice and patients.

	SD (%)	D (%)	N (%)	A (%)	SA (%)
**Dentists’ perception of the capability of teledentistry to improve practice**					
Teledentistry would provide an accurate diagnosis in a clinical setting	24 (3.7)	145 (22.2)	237 (36.3)	200 (30.7)	46 (7.1)
Teledentistry would help shorten the waiting list	0 (0)	9 (1.4)	45 (6.9)	408 (62.6)	190 (29.1)
Teledentistry would enhance guidelines and advice	1 (0.2)	20 (3.1)	112 (17.2)	401 (61.5)	118 (18.1)
Teledentistry would improve the interaction between peers	2 (0.3)	24 (3.7)	92 (14.1)	390 (59.8)	144 (22.1)
Teledentistry would provide a safe atmosphere for practicing dentistry	1 (0.2)	17 (2.6)	79 (12.1)	338 (51.8)	217 (33.3)
Teledentistry would make patients’ referrals more efficient	1 (0.2)	12 (1.8)	88 (13.5)	403 (61.8)	148 (22.8)
**Dentists’ perception of teledentistry usefulness for the dental practice**					
Teledentistry would enhance clinical training and continuing education	12 (1.8)	80 (12.3)	189 (29)	306 (46.9)	65 (10)
Teledentistry would reduce costs for the dental practices	1 (0.2)	54 (8.3)	182 (27.9)	334 (51.2)	81 (12.4)
Teledentistry would increase treatment time spent with the patient	12 (1.8)	150 (23)	224 (34.4)	209 (32.1)	57 (8.7)
Teledentistry would necessitate an extra appointment for taking photographs	1 (0.2)	39 (6)	102 (15.6)	422 (64.7)	88 (13.5)
Teledentistry would save time compared with a referral letter	0 (0)	18 (2.8)	62 (9.5)	402 (61.7)	170 (26.1)
Teledentistry would be too expensive to set up	11 (1.7)	189 (29)	307 (47.1)	129 (19.8)	16 (2.5)
Teledentistry would provide adequate diagnostic information	4 (0.6)	104 (16)	161 (24.7)	315 (48.3)	68 (10.4)
**Dentists’ perception of the usefulness of teledentistry for patients**					
Teledentistry would save money for patients	0 (0)	16 (2.5)	133 (20.4)	344 (52.8)	159 (24.2)
Teledentistry would improve communication with patients	2 (0.3)	14 (2.1)	74 (11.3)	384 (58.9)	178 (27.3)
Teledentistry would be helpful for patient education	6 (0)	28 (0.9)	389 (4.3)	389 (59.7)	229 (35.1)
Teledentistry would help to avoid unnecessary travel to the dental clinic	0 (0)	12 (1.8)	42 (6.4)	373 (57.2)	225 (34.5)
Teledentistry would be helpful in monitoring the patient's condition	1 (0.2)	13 (2)	54 (8.3)	435 (66.7)	149 (22.9)
Teledentistry would be convenient and well received by patients	3 (0.5)	23 (5.5)	239 (36.7)	289 (44.3)	85 (13)
Teledentistry would be useful for patients in remote areas	6 (0.9)	36 (3.5)	67 (10.3)	319 (48.9)	237 (36.3)
Teledentistry should be covered by dental insurance plans	4 (0.6)	17 (2.6)	119 (18.3)	301 (46.2)	211 (32.4)

SD, strongly disagree; D, disagree; N, neutral; A, agree; SA, strongly agree.

### Capability of teledentistry to improve practice

The domain consisted of 6 Likert-type questions that evaluate dentist perception of the capability of teledentistry to improve dental practice. This domain's mean score result in this study is 23.49 ± 3.001 of the total score, which could be as high as 35. The mean Likert score of this domain was 3.87 ± 0.53. Most of the respondents already realised the capability of teledentistry in improving dental practice, as shown in Table 2. The respondents either agree or strongly agree that teledentistry can shorten the waiting list, enhance guidelines and advice, improve peers’ interaction, provide a safe atmosphere in practice, and efficiently make patient referrals. However, only approximately one-third of respondents have positive agreement towards the ability of teledentistry to provide an accurate diagnosis.

### Usefulness of the teledentistry for patients

This domain consisted of 8 Likert-type questions that evaluate dentists’ perception of the usefulness of teledentistry for patients. This domain's mean score result in this study was 32.61 ± 3.96 of the total score, which was as high as 40. The mean Likert score of this domain was 4.07 ± 0.49. Most respondents were certain that teledentistry would benefit patients, as shown in Table 2. The results showed that most respondents believed that teledentistry would be useful for patients in remote areas, would be helpful in patient monitoring and education, reduced unnecessary travel, saved patients’ money, and improved communication. Slightly more than half of the respondents showed a positive perception that teledentistry can be well received by the patients. Most respondents also agreed that teledentistry should be covered by insurance.

### Dentists’ concerns regarding security and confidentiality issues

In relation to questions about concerns about teledentistry application, the highest number of little and significant concerns expressed by Indonesian dentists was related to the potential for digital forgery (74.2%) followed by hardware and software incompatibility (71.8%). The least concerning matter was gaining patient consent for teleconsultation (34.8%), as shown in Table 3.

**Table 3:** Dentists’ concerns about data security and patients’ consent.

	VC (%)	LC (%)	NA (%)	NP (%)	NC (%)
Gaining patient consent for teleconsultation	44 (6.7)	183 (28.1)	234 (35.9)	172 (26.4)	19 (2.9)
Confidentiality when data are sent online	126 (19.3)	317 (48.6)	133 (20.4)	71 (10.9)	5 (0.8)
Potential for digital forgery	107 (16.4)	377 (57.8)	110 (16.9)	54 (8,3)	4 (0,6)
Incompatible hardware and software	86 (13.2)	382 (58.6)	114 (17.5)	65 (10)	5 (0.8)
Reliability of teledental equipment	66 (10.1)	337 (51.7)	168 (25.8)	75 (11.5)	6 (0.9)

VC, very concerned; LC, little concerned; NA, not feeling either way; NP, not particularly concerned; NC, not concerned at all.

**Table 4:** Statistical analysis between dentists’ demographic variables of the respondents with the 4 domains of teledentistry dentists’ perception (Kruskal–Wallis test).

Variable	Dentists’ perception of the usefulness of the teledentistry for dental practice (3.32 ± 0.34)	Dentists’ perception of the capability of the teledentistry to improve practice (3.87 ± 0.53)	Dentists’ perception of the usefulness of the teledentistry for patients (4.07 ± 0.49)	Dentists’ concern about data security and patients’ consent (2.4 ± 0.65)
**Age (years)**				
20–34	3.31 (0.32)	3.83 (0.49)	4.03 (0.45)	2.39 (0.62)
35–44	3.31 (0.36)	3.88 (0.58)	4.10 (0.56)	2.41 (0.65)
45–54	3.37 (0.34)	3.94 (0.50)	4.12 (0.48)	2.39 (0.73)
55–64	3.35 (0.27)	3.91 (0.47)	4.12 (0.41)	2.49 (0.64)
≥65	3.43 (0.40)	3.60 (0.28)	3.75 (0.00)	2.30 (0.42)
*P* value	.743	.327	.141	.900
**Gender**				
Male	3.29 (0.35)	3.85 (0.59)	4.06 (0.54)	2.46 (0.73)
Female	3.33 (0.33)	3.87 (0.50)	4.08 (0.48)	2.39 (0.61)
*P* value	.344	.697	.957	.391
**Qualification**				
Specialists	3.31 (0.35)	3.81 (0.53)	4.13 (0.48)	2.42 (0.59)
General dentists	3.33 (0.33)	3.89 (0.53)	4.06 (0.49)	2.39 (0.66)
Residents	3.31 (0.35)	3.82 (0.50)	4.04 (0.49)	2.48 (0.64)
*P* value	.484	.089	.475	.587
**Work experience (years)**				
0–5	3.30 (0.34)	3.84 (0.52)	4.07 (0.44)	2.42 (0.63)
6–10	3.31 (0.33)	3.85 (0.51)	3.98 (0.54)	2.37 (0.62)
11–15	3.33 (0.36)	3.86 (0.55)	4.11 (0.49)	2.40 (0.63)
>15	3.35 (0.32)	3.92 (0.53)	4.15 (0.49)	2.42 (0.70)
*P* value	.702	.512	.039*	.914
**Location of main job**				
Not remote	3.32 (0.34)	3.85 (0.53)	4.08 (0.48)	2.41 (0.66)
Remote	3.37 (0.31)	3.92 (0.46)	3.99 (0.53)	2.37 (0.55)
Very remote	3.35 (0.51)	4.09 (0.73)	4.25 (0.67)	2.51 (0.66)
*P* value	.491	.257	.183	.848

⁎ Significant *P* value < .05 (Kruskal–Wallis test).

### Preferred dental specialty for teledentistry application

**Fig. 1:**
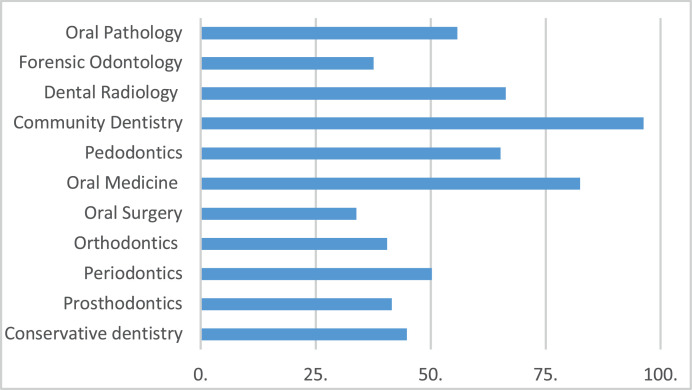
Dentists’ opinion related to the preferred dental specialty for application of teledentistry (%).

This study asked about the preferred dental specialty for teledentistry applications. In Indonesia, there are 11 recognised dental specialties. Community dentistry and oral medicine (82.5%) are the 2 specialties that were chosen for which teledentistry was mostly applicable. The specialty least preferred to be applied in teledentistry was oral surgery (33.9%) (Figure 1).

### Differences in the mean score of responses for demographic variables

**T**he Kruskal–Wallis test was performed to evaluate differences in the mean scores of responses for variables such as age, gender, working experience, qualification, and work setting of the main job. The dentists’ perception of the usefulness of teledentistry for dental practice, its capability to improve practice, and its usefulness for patients did not differ by most of the demographic characteristics of the dentists (*P* > .05; Table 4). Only the dentists’ working experience significantly altered their perception that teledentistry was useful for patients (*P* = .039).

## Discussion

The COVID-19 pandemic has expanded the use of telehealth, a variety of medical activities that are designed to support disease management and increase access to care, with the support of an extensive range of electronic communication technologies.^34^ Telehealth is also considered a useful and economical system for providing health care to underserved communities.^10^ Oral health is an essential part of general health; therefore, teledentistry is also applied in the same manner as telehealth.^34^ As a subunit of telehealth, teledentistry can be very beneficial to be applied to support access to care in dentistry, especially during this pandemic era.^24^ A meta-analysis conducted by Lin et al, regarding teledentistry recognition during COVID-19 pandemic studies, showed that dentists had a high level awareness and posititve attitute towards teledentistry.^35^ The positive awareness and attitude, however, are not in line with the lack of knowledge level or practice on teledentistry that might be attributed to conditions in different countries.^35^^,^^36^

This study collected data from dentists across Indonesia. To the best of our knowledge, this is the first study in Indonesia to explore dentists’ perceptions of the application of teledentistry in daily practice. This study also explored the determining factors that influence perception. The response rate might be a limitation to this study. The number of nonrespondents may have undermined the power of the study, although several reminders were sent to call for participating in the study. Furthermore, there may have been a response bias because the participants may only represent those who have a positive disposition to the study objective. However, the study received responses from dentists from 34 provinces in Indonesia. In general, this study showed that Indonesian dentists had a positive perception about the usefulness of teledentistry to improve practice and benefit patients compared to its advantages to dental practice. There were respondents who were concerned about some aspects of teledentistry practice. The lower perceptions of teledentistry used were related to diagnostic accuracy, time for taking clinical pictures, and cost. The dentists did not have strong views about whether teledentistry would increase the time spent with the patients.

Overall, the respondents exhibited positive perceptions of teledentistry's capability to improve dental practice, its utility for dental practice, and its potential to be highly beneficial for patients. There were no factors associated with perception, except dentists’ clinical experience. Similar responses were also collected from previous studies conducted in Australia and Saudi Arabia.^26^^,^^27^ Approximately one-third of respondents agreed on the statement that teledentistry would provide accurate diagnosis in a clinical setting; this result also corresponded with similar studies.^26^^,^^27^ This study showed that Indonesian dentists have a positive perception of the new regulation of teledentistry made by the Ministry of Health to provide services for patients in response to the circumstances of the COVID-19 pandemic. Other studies regarding teledentistry validity in diagnosis establishment have been conducted,^19^^,^^37^^,^^38^ but its application in Indonesia still needs further study.

Similar to dentists in other previous studies in Australia, Saudi Arabia, the UK, and Canada, Indonesian dentists were quite unsure about several aspects of teledentistry use.^13^^,^^26^^,^^27^^,^^39^^,^^40^ These aspects included cost related to setting up the clinic, extra technology or equipment used for teledentistry, time spent with the patient, and data security. These aspects of concern were actually similar to previous studies in Australia and Saudi Arabia although quite different from studies in the UK or Canada that indicated that remuneration, technology, and security issues do not cause as much concern.^13^^,^^26^^,^^27^^,^^40^ This result indicated that if dentists practice teledentistry, they should invest maximum efforts to protect the confidentiality of related information. The system that is adopted for teledentistry should be able to secure patients’ data confidentially in an electronic form.^41^

The demographic characteristics of this study showed that the majority of Indonesian dentists were female, which was very different from similar studies in which the majority of participants were male dentists.^26^^,^^27^ Whilst a previous study in Australia found that dentists preferred email and phone, the dentists in this study highly preferred the use of social media, such as WhatsApp, to be specific, which was similar to a study in Saudi Arabia.^26^^,^^27^ Currently, communication applications have been encrypted to address concerns related to data privacy.^42^^,^^43^ As a popular smartphone application, WhatsApp has been widely used in Indonesia, so it was chosen as a medium in distributing questionnaires in this study. WhatsApp has also been studied as a telemedicine platform in the oral medicine field.^44^ The use of this application as well as an Android-type smartphone may be the possible teledentistry preferred tools to be used in Indonesia.

Further concerns of teledentistry use were related to the ability to make accurate diagnoses. In several areas of dentistry, tele-surveys have been used to screen for dental caries and periodontal diseases.^45^^,^^46^ However, in some areas of dentistry, the uncertainty of diagnostic accuracy may be related to the inability to perform clinical examinations other than visualisation to establish an accurate diagnosis.^47^

The dentists in this study thought that teledentistry could be implemented in many specialties in dentistry. Community dentistry and oral medicine were the 2 fields that had the highest agreement about the application of teledentistry by the respondents. Many studies have been performed to explore the potency of teledentistry, especially in dental screening in the community.^45^^,^^48^^,^^49^ The application of teledentistry in oral medicine has been supported by some studies conducted in various countries.^19^^,^^23^^,^^44^^,^^50^ This positive opinion is very important since, in a pandemic setting, there are many situations that limit dentists in treating patients in dental clinics.^24^

A study amongst a small number of patients on their satisfaction on the application of teledentistry during the COVID-19 pandemic showed positive results.^51^ This study has added the perspective of teledentistry from the dentists’ point of view. However, further study on teledentistry usage both from the patients' point of view and from dentists’ knowledge and practice in Indonesia should be performed. The application of teledentistry in Indonesia is very promising for implementation in many areas of dentistry, depending on the type of treatment needed by the patients. Teledentistry would provide a good method to improve patient dental care in terms of preventing longer waiting times and treatment delays.^19^

## Conclusions

As the government has encouraged the use of telemedicine in Indonesia in response to the pandemic, Indonesian dentists have expressed their positive perception of the usefulness of teledentistry to improve practice and benefits for patients. Several concerns are related to diagnostic accuracy, cost, technology, and data security. These concerns indicate that when teledentistry is implemented widely in Indonesia, support and regulation should be clearly defined by policy makers. Clear standards and regulation of teledentistry would ensure benefits for dentists in providing patient care in daily practice.

## Appendix Supplementary materials

Image, application 1
