# Supplementary material for: Indonesian Dentists’ Perception of the Use of Teledentistry
Source: Int Dent J. 2022 May 12;72(5):674–81. doi: 10.1016/j.identj.2022.04.001 (PMC9485514; doi:10.1016/j.identj.2022.04.001)
Supplement: Supplementary file 1 [file mmc1.pdf]

ELSEVIER

Certificate of Elsevier Language Editing Services

The following article was edited by Elsevier Language Editing Services:

"Indonesian Dentists' Perception Toward the Use of Teledentistry"

Authored by:

Yuniardini S Wimardhani, Anandina I Soegyanto

Date: 29-Jul-2021

Serial number: LE-217356-99F992271EAB
